# Prenatal alcohol exposure is a risk factor for adult neuropathic pain via aberrant neuroimmune function

**DOI:** 10.1186/s12974-017-1030-3

**Published:** 2017-12-19

**Authors:** Joshua J. Sanchez, Shahani Noor, Suzy Davies, Daniel Savage, Erin D. Milligan

**Affiliations:** 1 Department of Neurosciences, School of Medicine, University of New Mexico Health Sciences Center, Albuquerque, NM 87131-0001 USA; 2 Department of Anesthesiology and Critical Care Medicine, University of New Mexico Health Sciences Center, MSC08 4740, Albuquerque, NM 87131-001 USA

**Keywords:** Neuropathic pain, Prenatal alcohol exposure, Glia, Neuroimmune function, Peripheral immune system, Spinal cord

## Abstract

**Background:**

Clinical studies show that prenatal alcohol exposure (PAE) results in effects that persist into adulthood. Experimental animal models of moderate PAE demonstrate that young adults with PAE display potentiated sensitivity to light touch, clinically termed allodynia, following sciatic nerve chronic constriction injury (CCI) that coincides with heightened spinal glial, spinal macrophage, and peripheral immune responses. However, basal touch sensitivity and corresponding glial and leukocyte activation are unaltered. Therefore, the current study explored whether the enduring pathological consequences of moderate PAE on sensory processing are unmasked only following secondary neural insult.

**Methods:**

In middle-aged (1 year) Long Evans rats that underwent either prenatal saccharin exposure (control) or moderate PAE, we modified the well-characterized model of sciatic neuropathy, CCI, to study the effects of PAE on neuro-immune responses in adult offspring. Standard CCI manipulation required 4 chromic gut sutures, while a mild version applied a single suture loosely ligated around one sciatic nerve. Spinal glial immunoreactivity was examined using immunohistochemistry. The characterization and functional responses of leukocyte populations were studied using flow cytometry and cell stimulation assays followed by quantification of the proinflammatory cytokines interleukin-1beta (IL-1β) and tumor necrosis factor-alpha (TNF-α). Data were statistically analyzed by ANOVA and unpaired *t* tests.

**Results:**

The current report demonstrates that mild CCI generates robust allodynia only in PAE rats, while the pathological effects of PAE following the application of a standard CCI are revealed by enhanced allodynia and elevated spinal glial activation. Additionally, mild CCI increases spinal astrocyte activation but not microglia, suggesting astrocytes play a larger role in PAE-induced susceptibility to aberrant sensory processing. Leukocyte populations from PAE are altered under basal conditions (i.e., prior to secondary insult), as the distribution of leukocyte populations in lymphoid organs and other regions are different from those of controls. Lastly, following in vitro leukocyte stimulation, only PAE augments the immune response to antigen stimulation as assessed by heightened production of TNF-α and IL-1β.

**Conclusions:**

These studies demonstrate PAE may prime spinal astrocytes and peripheral leukocytes that contribute to enduring susceptibility to adult-onset neuropathic pain that is not apparent until a secondary insult later in life.

## Background

The adverse effects of alcohol consumption on the developing central nervous system (CNS) have been recognized for decades and include an overt and distinct pattern of malformations that occur following the highest exposure levels, referred to as fetal alcohol syndrome [1, 2]. However, it is now recognized that a wide range of disabilities are associated with much lower levels of alcohol exposure during fetal development resulting in a spectrum of CNS-related disorders. This spectrum of disorders is referred to as fetal alcohol spectrum disorder (FASD) [2–4]. Associated disabilities that occur across the lifespan as a result of moderate exposure to alcohol during CNS development include not only behavioral and cognitive deficits [4–6], but also sensory touch abnormalities in individuals with FASD [7]. In support of these clinical observations, preclinical experiments in adult rhesus monkeys reveal touch abnormalities such as tactile hypersensitivity are observed in monkeys exposed prenatally to low/moderate levels of alcohol [8].

Tactile hypersensitivity, referred to as light-touch mechanical allodynia frequently observed in chronic pain patients, is caused by spinal cord neuronal alterations that pathologically convert non-painful touch into painful signals [9]. Anatomically, sensory peripheral nerve terminals project to the spinal cord and communicate to these pain-responsive neurons in the spinal cord [10]. Notably, allodynia often occurs following damage to peripheral nerves [11]. Studies using animal models of neuropathic pain, such as a standard localized sciatic nerve damage referred to as chronic constriction injury (CCI), have demonstrated that spinal immune and glial cells (astrocytes and microglia) release proinflammatory cytokines such as interleukin-1β (IL-1β) and tumor necrosis factor-α (TNF-α), which play critical roles in mediating chronic allodynia [10, 12–14]. A number of reports accumulating for several decades show that animal models of chronic peripheral neuropathy develop pathological pain through aberrant neuroimmune mechanisms (i.e., activated glia and proinflammatory cytokines) [10, 14].

Similarly, animal models of prenatal alcohol exposure (PAE) have advanced our understanding of the pathophysiology caused by mild to moderate levels of alcohol exposure and some support possible aberrant neuroimmune actions underlying CNS-related pathologies that persist into adulthood [15]. For example, experimental rodent PAE models show increased reactive brain microglial and astrocyte responses [15, 16], elevated brain proinflammatory cytokine and chemokine production [16–19], decreased microglial survival [15], and altered brain glial and immune responses following injury [20–22]. Notably, these data support clinical observations of patients with FASD that also present with aberrant immune responses such as augmented lymphocyte formation [23] and altered cytokine synthesis [24].

More recently, rat offspring with moderate levels of PAE reveal potentiated tactile hypersensitivity (a.k.a. allodynia) following adult-onset peripheral nerve damage that occurs in parallel with augmented spinal glial activation and peripheral immune responses, while no such changes are observed in non-alcohol exposed rodents [25]. These data suggest PAE-induced aberrations in neuroimmune function may underlie the clinical sensory abnormalities observed in individuals with FASD. Moreover, the most intriguing aspects of the data are that PAE alone is insufficient to alter basal touch sensitivity. Specifically, the adverse effects of PAE on abnormal sensory processing and associated spinal glial and immune function only become apparent following a secondary insult, which, in these experiments, is the application of a well-characterized CCI nerve injury model [25]. These data suggest that moderate PAE may create susceptibility for developing neuropathic pain that occurs long into adulthood. Therefore, the hypotheses of the current set of studies are twofold: (1) that the damaging effects of PAE on the CNS are unmasked only following an insult or challenge to the peripheral/central nervous system and (2) the magnitude of the response to the insult is disproportionately greater to the degree of the insult itself. The goal of these studies is to determine that PAE renders one susceptible to neuropathic pain induced by mild sciatic nerve injury, with PAE-induced neuropathy mediated by exaggerated alterations in spinal glial and immune responses.

Applying a well-characterized rodent model of sciatic neuropathy in adult offspring with PAE provides insight into understanding (1) aberrant immune cell and glial CNS activation as a consequence of PAE and (2) adverse conditions during CNS development that create adult susceptibility to developing chronic neuropathic pain from minor or mild insults. Reducing injury to the sciatic nerve from the well-characterized standard 4-suture to a minor 1-suture CCI addresses two critical questions. First, while the standard sciatic nerve CCI in the rat is well-characterized to involve spinal glial activation and other trafficking leukocytes [10, 26–28], no reports exist showing 1-suture CCI generates allodynia. Second, the chronic underlying pathology from PAE on CNS sensory processing is best unmasked following mild injury. Thus, the goals of the current report sought to unmask the effects of PAE on augmented spinal glial (astrocyte and microglial) phenotypes and profiles, and functional responses of peripheral immune cells characterized to traffic to the spinal cord to identify aberrant allodynia and neuroimmune reactivity.

## Methods

### Animals and study group strategy

All procedures were approved by the Institutional Animal Care and Use Committee (IACUC) of The University of New Mexico Health Sciences Center and closely adhered to guidelines from the International Association for the Study of Pain for the use of animals in research. Long-Evans rat breeders purchased from Harlan Industries (Indianapolis, IN) were maintained in a breeding colony on a 12:12-h reverse light/dark schedule (lights on from 2100 to 0900 h), and fed standard rat chow and water, available ad libitum. For all experiments, 12–13 month male prenatal alcohol or saccharin exposed (described below) rat offspring derived from 14 litters were used. Offspring were habituated to a standard light/dark cycle (lights on from 0600 h to 1800 h) for at least 5 days and kept in these conditions for the duration of the study. A total of 14 female Long-Evans rat dams and 50 male offspring were used in all experiments. Offspring (12–13 months of age) were predominantly pair housed with 5 singled housed due to incompatibility with a cage mate.

### Moderate prenatal alcohol exposure using the voluntary drinking paradigm

Pregnant female rat dams were given either ethanol or saccharin throughout pregnancy until birth according to the voluntary drinking paradigm previously described [29]. Briefly, 3- to 4-month female Long-Evans rat breeders were acclimated for 4 h per day, from 1000 to 1400 h, to drinking water containing 0.066% (*w*/*v*) saccharin (Sac) that gradually increased in ethanol content from 0% (*v*/*v*) on days 1–2, to 2.5% (*v*/*v*) on days 3–4, to 5% (v/v) on day 5 and thereafter for 2 weeks. It should be noted that regular drinking water is available at all times during housing. Thus, animals could voluntarily choose to drink either saccharin-sweetened water containing 5% ethanol or regular water during the 4-h drinking period. At the end of the 2-week prepregnancy drinking phase, the mean daily ethanol consumption was determined for each rat and rats that consumed 1 standard deviation above or below the group mean were excluded from the study. Subsequently, female rats were assigned to either a 5% ethanol or Sac control drinking group such that the mean prepregnancy ethanol consumption was similar between groups. The females were then placed with proven male rat breeders until pregnant. No alcohol was consumed during the breeding period, which averaged between 1 and 2 days. Beginning on gestational day 1, rat dams were given either 0% (Sac) or 5% (PAE) ethanol in Sac water (4 h/day). Sac control group rats were given a volume of 0% ethanol in Sac water that was matched to the mean volume voluntarily consumed by the 5% ethanol group. Total ethanol consumption was recorded for each dam, which averaged 2.04 g of ethanol/kg body weight/day. This level of drinking by rat dams produced a mean peak serum blood alcohol concentration of 0.06 g/dl. No significant differences were observed between prenatal treatment groups in dam weight gain during pregnancy, pup birth weights, or litter size, replicating previous reports [29]. Offspring were weaned at 24 days of age and male offspring pair-housed with the exception of five single-housed rats due to incompatibility with a cage mate. For all experiments, 12- to 13-month prenatal alcohol or saccharin-exposed animals were used. A total of 50 male offspring were used in these experiments. Behavioral testing of offspring was performed during the first 3 h of the light cycle to avoid the influence of elevated hormones under normal circadian rhythms.

### Chronic constriction injury (CCI)

In adult (12–13 months) male Sac or PAE rats, sham or CCI aseptic surgical procedures were performed as previously described [25, 28] and modified in some experimental groups, as described here. Under isoflurane anesthesia (induction 5% vol. followed by 3.5% in oxygen), the sciatic nerve was carefully isolated with sterile glass prongs and snuggly ligated with 1 (minor/mild injury) or 4 (standard injury; [28]) segments of sterile 4–0 chromic gut sutures (Ethicon, Somerville, NJ) without pinching into the nerve. Sterile isotonic saline (0.9%) was applied to the nerve during the procedure to prevent dehydration. Sham surgery involved isolation of the sciatic nerve similar to CCI but without nerve ligation. The nerve was then gently placed back into position and the overlying muscle was sutured closed with two 3–0 sterile silk sutures (Ethicon, Somerville, NJ). Rats fully recovered from anesthesia within approximately 5 min and were monitored daily following surgery for any post-operative complications.

### Behavioral assessment of allodynia

Allodynia was assessed using the von Frey fiber test as previously described [27]. Briefly, rats were first habituated to the testing environment by placing rats atop 2-mm thick parallel bars spaced 8 mm apart allowing full access to the plantar hindpaw. Habituation occurred for approximately 45 min/day for 4 sequential days within the first 3 h of the light cycle in a sound- and temperature-controlled dimly lit section of the colony room. Baseline responses were then assessed using the von Frey behavioral test. For these experiments, the scale range of the calibrated monofilaments was modified and included 3 monofilaments calibrated to bend at 0.027 g (2.44 log stimulus), 0.067 g (2.83 log stimulus), and 0.166 g (3.22 log stimulus). Thus, a total of 13 calibrated monofilaments were used in these studies. Additionally, these calibrated monofilaments (2.44–5.18 log stimulus intensity) were applied randomly to the plantar surface of the left and right hindpaw for a maximum of 8 s per application. A metronome placed in the room provided guidance at 1 tick/s. Lifting, licking, or shaking of the paw was considered a response. In a similar manner to baseline evaluation, animals were re-assessed following CCI or sham surgery on days 3 and 10. The experimental tester was blind to the prenatal and surgical treatment groups.

### Immunohistochemical (IHC) tissue sample preparation

All tissue was collected from behaviorally verified rats on day 10 after sham or CCI surgery. Tissues were processed as described previously [25]. Briefly, rats were overdosed with sodium phenobarbital (Sleepaway, Fort Dodge Animal Health, Fort Dodge, IA) and transcardially perfused with 0.1 M phosphate buffered saline (PBS; pH = 7.4), initially at a rate of 28 ml/min, then increased to 32 ml/min (~ 10 min), followed by 4% paraformaldehyde (PFA; pH = 7.4; 28–34 ml/min, 8 min). Immediately following transcardial perfusion, the spinal vertebral column from C2-L6 with the spinal column intact within the vertebral column were collected and cut at vertebra T7 into a rostral and caudal half of the spinal vertebral column. All spinal cord sections underwent 48-h post-fixation in 4% PFA at 4 °C. Approximately, a third (initial *N* = 12) of the total spinal cords collected for IHC underwent decalcification. The purpose for maintaining the spinal cord within the vertebral column was to identify possible immune cell and astrocytic endfeet changes that occur within the subarachnoid matrix known to house a sentinel and infiltrating immune cells. Our prior work employing this method documents our success in younger rats (3- to 4-month-old rats) [26, 30]. Decalcified spinal cords underwent unexpected prolonged tissue decalcification, possibly due to the age of the rats (1-year olds in the current study) that resulted in altered integrity of the Iba1 epitope as virtually no IR signal was detectable. To avoid this problem, spinal cords (*N* = 24) collected from subsequent experiments did not undergo decalcification and were isolated as described below. Thus, each experimental condition consisted of sample sizes of *N* = 6 rats.

For spinal vertebral columns that underwent decalcification, segments were placed in a 3-l water bath containing 10% ethylenediaminetetraacetic acid (EDTA) (Sigma-Aldrich, St. Louis, MO), 0.01% sodium azide, and 0.5% paraformaldehyde with gentle consistent stirring atop a stir plate, with the solution exchanged every 5 days. A fully decalcified spinal vertebral column was determined when no resistance occurred upon penetrating the dorsal and lateral spinous processes with a 30-gauge hypodermic needle. At 4 months of decalcification, spinal cords were isolated from vertebral columns.

For non-decalcified and decalcified vertebral columns, entire spinal cord (T7-L6) was carefully isolated from the spinal vertebral column making sure to maintain an intact spinal cord. The isolated spinal cords were segmented and subsequently paraffin processed. L4-L5 paraffin-embedded blocks were subsequently sliced on a microtome with adjacent 7-μm tissue sections mounted onto vectabond-treated slides.

To investigate spinal augmented glial activation, we analyzed the expression of the astrocyte marker, glial fibrillary acidic protein (GFAP), and the microglial activation marker ionized calcium-binding adapter molecule 1 (Iba1) in L4-6 spinal segments reported previously [26]. In addition, we analyzed the expression of the microglial specific marker, transmembrane protein 119 (TMEM119) as described below. Slides containing tissue from lumbar (L4-L6) spinal cord were chosen for glial staining to capture overall glial reactivity [26]. Paraffin-processed tissues underwent deparaffinization followed by rehydration and antigen retrieval procedures in a rice cooker at 94–96 °C allowing for a gentle and equal distribution of temperature warming throughout tissue sections. For antigen retrieval, GFAP required a Tris-based buffer at pH 9.5 (BioCare Medical, Concord, CA), Iba1 required a Tris-based buffer at pH 9.0 (Vector, Burlingame, CA), and TMEM119 required a citrate-based buffer at pH 6.0 (BioCare Medical, Concord, CA). All tissue sections were incubated with 5% normal donkey serum (NDS), PBS pH 7.4 for 2 h, followed by overnight primary antibody incubation using rabbit anti-rat GFAP (Millipore, 1:1000) [26], rabbit anti-rat Iba-1 (Wako, 1:300) [26], or rabbit anti-rat TMEM119 (Abcam, 1:100) [31] in a humidity chamber at 4 °C. Tissues were washed 3× with 0.1 M PBS pH 7.4 followed by donkey-anti rabbit FITC- or TRITC-conjugated secondary antibody incubation for 2 h in a humidity chamber at room temperature, and rinsed in 0.1 M PBS. Tissues were then stained with the nuclear stain 4,6-diamidino-2-phenylindole (DAPI) (Vector Labs, Burlingame, CA) separately before cover slipping. These slides were left at room temperature overnight before proceeding with image acquisition and microscopy analysis.

### Microscope spectral imaging for immunofluorescent quantification

Image acquisition for spectral analysis was performed using the Nuance spectral imaging system (http://www.cri-inc.com/products/nuancew.asp; Perkin Elmer, Waltham, MA) as described previously [25]. Briefly, images of dorsal horn spinal cord were obtained using a 20x objective with a Zeiss Axioplan2 inverted fluorescence microscope. Flat-field correction was applied in order to produce a uniform illumination during image acquisition. Image cubes were obtained from multi-labeled tissue containing DAPI, conjugated secondary antibody, as well as autofluorescence. A spectral library was then created using single-labeled slides for each fluorophore (e.g., FITC or TRITC) and a label-free (autofluorescence) slide. A computed spectrum (420–720 nm) was obtained by separating the known spectrum (autofluorescence) from mixed spectrum (single labeled) to produce pure spectrum of each fluorophore. This allowed the separation of spectrum from multi-labeled slides (e.g., DAPI + TRITC) to obtain composite images containing only labels of interest. These composite images were then used for further analysis using Slidebook 6 software (below). Twelve to 24 images per experimental group (4 sections per animal, 3–6 animals per condition) per side (ipsilateral and contralateral) of sciatic manipulation were acquired and analyzed. In addition, the Nuance spectral imaging system was used for representative acquired images of TMEM119 shown in Fig. 3e. 20x and 60x objectives were used to produce representative images.

### Slidebook software image analysis

Composite images were analyzed using Slidebook 6 software (Intelligent Imaging Innovations, Denver, CO, USA). To eliminate signals originating from artifacts, the experimenter determined an acceptable threshold of very low-level emission fluorescent intensity per each experimental condition (e.g., Sac + Sham, PAE + Sham) by closely replicating the on-screen composite computer image with that observed through the microscope eyepiece, as described previously [25]. In addition, the dorsal horn of the spinal cord (region of interest (ROI)) was outlined for analysis eliminating the surrounding white matter and peri-spinal blank space (intrathecal space). The image was then refined to include the predetermined threshold within the outlined area. The “sum intensity” (the total signal within the outlined area in the dorsal horn) was determined and divided by the “area” (total area in micrometers squared (μm^2^)) to obtain the “fluorescence intensity”. The average of four adjacent sections from a single slide (representing a single animal) was calculated to determine the value for each animal. Finally, averages for each slide within the same condition were calculated along with the standard deviation and standard error of the mean. Data are analyzed as “sum intensity of ROI/μm^2^”. Fluorescence intensity was characterized from these thin sections to capture staining density and intensity of both astrocytes and microglia at the dorsal lamina (I–V) within a single lumbar segment to assess the overall highly localized profile, as both GFAP and Iba1 are well-documented to upregulate these markers upon increased cellular activation [32, 33].

### Preparation of single-cell suspensions of peripheral immune cells for flow cytometry

Surgically naive rats (Sac-control and PAE) were deeply anesthetized with Isoflurane (Piramal Healthcare, Mumbai, India) (8–10 min, 5% vol in oxygen). Peritoneal exudate cells (PECs), peripheral blood, thymus, spleens, and medial iliac lymph nodes were collected and immediately placed on ice. Medial iliac lymph nodes are draining lymph nodes for sciatic nerves, and the immune cells from these lymph nodes were examined [34, 35]. Single-cell suspensions from PECs, spleens, and peripheral blood were prepared identically, as described previously [25]. Briefly, PECs were collected using ice-cold Iscove’s media (Sigma-Aldrich, St. Louis, MO) and pelleted by centrifuge at 300×*g* at 4 °C for 8 min. The resultant cell pellet was incubated with 2 ml hypotonic salt solution (ACK lysis buffer; Sigma-Aldrich, St. Louis, MO) for 5 min on ice to lyse red blood cells (RBCs). One million cells were resuspended in 2 ml ice-cold 0.1 M PBS (pH = 7.4) and processed for flow cytometry as described here. The remaining cells were used for in vitro stimulation experiments described below.

Spleens were harvested in RPMI 1640 media (medium originally developed by Roswell Park Memorial Institute, purchased from Sigma-Aldrich, St. Louis, MO) and homogenized via passage through a 40-μm cell strainer (Corning™ sterile cell strainers, Fisher Scientific, USA) to prepare a single-cell suspension. RBCs were lysed similarly to that conducted with PECs. Splenocytes were used for flow cytometry described below. Single-cell suspensions from lymph nodes and thymus were prepared using 40-μm cell strainers, conducted similarly to the preparative procedures described for isolating splenocytes. However, the RBC lysis step was not performed for lymph node and thymus samples.

Peripheral blood was collected (in BD vacutainer^R^ K_2_EDTA blood collection tube) through cardiac puncture immediately after PECs collection. Peripheral blood (PBMNs) were isolated using Ficol Premium 1.84 (GE Healthcare Life Sciences, PA, USA) according to the manufacturer’s instructions. Briefly, 1-ml blood was diluted to 4 ml with PBS (w/o Ca/Mg) and layered on 3 ml Ficol in a 15-ml conical tube and centrifuged at 400×*g* for 30 min at 20 °C, without brakes. PBMNs were collected from the interface and washed twice with PBS at 400×*g* for 10 min at 20 °C. Cells were resuspended in 2 ml of 0.1 M PBS pH 7.4 on ice until proceeding to identify live cells (described below; viability dye staining).

### Flow cytometry data acquisition and analysis of immune cell profiles in PAE rats

Using flow cytometry, major immune cell subtypes in Sac and PAE rats were identified using the following gating strategy: live cells were identified based on the absence of cell viability dye, B and T cells contained positive CD3 and CD45RA expression respectively, NK cells contained positive CD161 expression, and myeloid cells were positive for CD11b/c expression.

Live cells were counted on a hemocytometer using the trypan blue staining exclusion criteria. Between 0.5 × 10^6^ and 1 × 10^6^ cells were transferred in a FACS tube (BD Falcon™, MA, USA) and pelleted by centrifugation at 300×*g* for 5 min at 4 °C, with the supernatant discarded. Cells were then resuspended in PBS (without calcium and magnesium; Sigma-Aldrich, St. Louis, MO) and stained with Viability Dye eFluor® 450 (eBioscience, San Diego, CA) for 30 min, washed with FACS buffer (×1 PBS containing 1.0% bovine serum albumin, and 1 mM EDTA) and incubated with a saturating solution of Fc block (BD Biosciences, San Jose, CA, USA) for 10 min followed by staining with fluorochrome-conjugated antibodies for 30 min. All of these steps were conducted on ice. Antibodies against rat CD3 (cluster of differentiation 3, clone IF4) and CD45 RA (clone OX-33) were purchased from BD Biosciences (San Jose, CA, USA). CD11b/c (clone OX-42, recognizes a common epitope shared by Integrin αM and αX chains) and CD161 (clone 10/78) were purchased from Affymetrix (Santa Clara, CA, USA). These antibodies were used for 0.125–0.5 μg/10^6^ cells, as recommended by the manufacturer. Following antibody staining, cells were washed and resuspended in 300 μl FACS buffer and then passed through a 40-μm cell strainer immediately prior to analysis to avoid cell clumping. At least 50,000 live cell events were collected for each sample. Data were acquired using the BD LSR Fortessa cell analyzer (BD Biosciences, San Jose, CA) and analyzed using FlowJo software v.8.7.4 (Treestar Inc., Ashland, USA). Live, intact healthy cells were identified based on their size (Forward Scatter (FSC-A)) versus granularity (Side Scatter (SSC-A)). Dead cells were further excluded by the presence of positive viability dye staining and only cells displaying the absence of viability dye were included for flow cytometry analysis for different immune markers. T cell and B cell proportions were determined by positive expression of CD3 (T cell co-receptor) and CD45 RA (CD45 isoform expressed by only B cells) respectively. Natural killer cells (NK) were identified by negative expression of T/B cell markers and positive expression of CD161 (expressed predominantly on NK cells and involved in activating NK-cell-mediated toxicity [36, 37]). Myeloid cells (e.g., macrophages, monocytes, dendritic cells, and neutrophils) were identified by positive expression of CD11b/c and negative expression for lymphoid cell (T cell, B cells, and NK cells) markers.

### Intracellular detection of TNFα from splenocytes by flow cytometry

To examine the production of the proinflammatory cytokine tumor necrosis factor-α (TNFα), splenocytes collected from each rat were stimulated in vitro*,* as described previously [25]. Briefly, splenocytes were collected from rats and resuspended in RPMI 1640 complete medium supplemented with 10% (*v*/*v*) fetal bovine serum (FBS) (Sigma), 2.0 mM l-glutamine (Thermos Fisher Scientific, PA, USA), 50 μM 2-mercaptoethanol (Sigma-Aldrich), 100 U/ml penicillin and 100 μg/ml streptomycin (Thermos Fisher Scientific, PA, USA) to a cell density of 1 × 10^6^/ml. Splenocytes were plated in a 24-well culture plate (Corning Costar, Sigma-Aldrich) 1 × 10^6^ cells/well, and each well was stimulated with 50 ng PMA (a phorbol ester, Protein Kinase C activator, 50 ng/ml) and ionomycin (a calcium ionophore, 1 μg/ml), incubated for 5 h at 37 °C and 5% CO_2_. In order to block secretion of cytokines from activated splenic leukocytes, 2 μl/ml protein transport inhibitor cocktail (containing brefildin A and monensin, from eBioscience) was simultaneously added with PMA/ionomycin (PMA/Io) solution at the beginning of the cultures. Following 5 h of stimulation, splenic cells were gently removed from the wells, washed twice with PBS, and stained with viability dye and surface marker CD11b/c, as described above. Cells were fixed with 4% PFA (Sigma-Aldrich) for 10 min at room temperature. Following fixation, cells were permeabilized with 0.3% saponin (Sigma-Aldrich) in FACs buffer followed by incubation with anti-rat TNFα at 1 μg/10^6^ cells (Affymetrix, Santa Clara, CA, USA) for 40 min on ice in the dark. Cells were then washed twice in saponin-FACs buffer and processed for flow cytometric data acquisition. Viable cells were identified as described above. Cd11b/c cells expressing TNFα are represented. Proportions of TNFα^+^ leukocytes were evaluated based on the isotype control staining.

### IL-1β and TNF-α detection by enzyme-linked immunosorbent assay (ELISA)

PECs from each animal were plated in a 24-well tissue culture plate at 2 × 10^5^ cells per well, in duplicate followed by stimulation or control treatment. Cells were stimulated with 1 μg/ml lipopolysaccharide at 37 °C (LPS, major cell-membrane component from gram-negative bacteria) purchased from Sigma-Aldrich, St. Louis, MO, USA. LPS (diluted in RPMI). Following a 24-h stimulation, plates were spun at 300×*g*, 5 min and cell-free supernatants collected and stored at − 80 °C until assaying. Cells were washed 2× with ice-cold PBS. PBS was aspirated, and cell lysates were prepared using a whole cell lysis buffer (Pierce^R^ IP lysis buffer, Thermo Fisher Scientific, Waltham, MA, USA). Briefly, for each well, cells were treated with 120 μl of cell lysis buffer, on ice, for 10 mins. To remove cell debris, cell lysates were transferred to microcentrifuge tubes and spun at 13,000×*g*, 10 min, 4 °C. Cell lysates were collected and stored at − 80 °C, avoiding freeze/thaw cycles. TNFα and interleukin 1β (IL-1β) levels were measured using commercially available ELISA kits (R&D Systems, Minneapolis, USA). Each experimental well (from the tissue culture plate) was run in duplicate on the 96-well ELISA plate. All experiments and data analysis were performed according to the manufacturer’s instructions.

### Statistical analysis

SPSS (IBM, Chicago, IL, USA) was used for all behavioral analysis. At BL, a two-way (2 × 3) analysis of variance (ANOVA) was used for analysis of the between-subject factors of prenatal exposure (Sac versus PAE) and surgery (Sham versus minor CCI versus Standard CCI). Additionally, a two-way (2 × 3) repeated measures ANOVA was used for analysis of the between-subject factors of prenatal exposure and surgery for days post-surgery. Data from acquired microscope images, flow cytometry, and ELISA were analyzed using GraphPad Prism version 7 software (Graphpad Software Inc., San Diego, CA, USA). IHC IR was analyzed using a two-way (2 × 3) ANOVA followed by Fisher’s LSD test for post hoc examination. A total of *N* = 6; Sac and *N* = 8; PAE rats were used for characterizing peripheral immune cell phenotypes and their functional responses. To ensure healthy “live” single-cell suspension and immediate flow cytometry data acquisition (without fixation), rats were equally divided into two different tissue collection days (*N* = 3/day; Sac and *N* = 4/day; PAE, per experiment yielding a total of 5 different tissue samples per animal), which is a standard number of animals used in flow analysis [38–41]. Each tissue digestion and flow cytometry data collection day was considered an independent biological experiment. Data from each experiment was combined to generate *N* = 6 for Sac and *N* = 8 for PAE. Data comparing surgically naïve Sac and PAE groups were analyzed with unpaired *t* tests. ELISA data for pro-inflammatory cytokine production were analyzed using two-way (2 × 2) ANOVA. To control the type I error rate during multiple comparisons, reported with adjusted *P* values, Tukey’s test was applied for post hoc examination. The threshold for statistical significance was set a priori at *α* = 0.05 for all sets of multiple comparisons. In order to minimize unnecessary duplication, we used the minimum number of animals possible to make statistically significant conclusions, which was based on our previous publications [25–27, 42]. These studies utilized sample sizes of *N* = 3 (IHC analysis) and *N* = 6 (behavioral analysis) to reliably produce statistically significant differences between treatment groups. Outliers were removed following Grubbs’ Z-test [43]. In all cases, the data are presented as the mean ± SEM.

## Results

### PAE induces long-lasting susceptibility to allodynia from even minor peripheral nerve damage

**Fig. 1:**
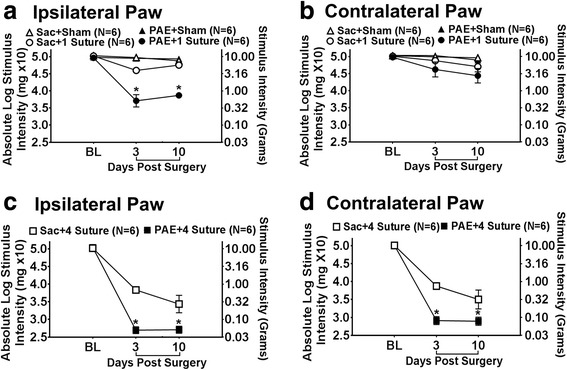
Prenatal alcohol exposure (PAE) causes *enhanced* allodynia following standard sciatic damage and allodynic *susceptibility* following minor nerve damage. Hindpaw response thresholds from (**a**, **b**) minor (1-suture) vs (**c**, **d**) standard (4-suture) unilateral sciatic nerve injury (CCI) in middle-aged PAE rats. **a**–**d** All Sac and PAE rats displayed similar baseline hindpaw sensitivity responses following application of mechanical stimuli [ipsilateral, F_2,30_ = 1.877, p = 1.102; contralateral, F_2,30_ = 0.409, p = 0.668]. Following surgical sciatic nerve manipulation, a main effect of alcohol exposure [ipsilateral, F_1,30_ = 56.721, p < 0.0001; contralateral, F_1,30_ = 11.868, p = 0.002], surgery [ipsilateral, F_2,30_ = 155.795, p < 0.0001; contralateral, F_2,30_ = 99.261, p < 0.0001], and an interaction between alcohol exposure and surgery was seen [ipsilateral, F_2,30_ = 13.785, p < 0.0001; contralateral, F_2,30_ = 4.860, p = 0.015]. **a**, **b** Following minor CCI, PAE rats developed increased sensitivity whereas Sac rats did not. A main effect of alcohol exposure [ipsilateral, F_1,30_ = 61.875, p < .0001; contralateral, F_1,30_ = 12.857, p = 0.001], surgery [ipsilateral, F_2,30_ = 180.372, p < 0.0001; contralateral, F_2,30_ = 108.621, p < 0.0001], as well as an interaction between alcohol exposure and surgery was revealed [ipsilateral, F_2,30_ = 1.716, p < 0.0001; contralateral, F_2,30_ = 5.276, p = 0.011]. Asterisks indicate *p* < 0.05. The data are presented as the mean ± SEM

Under basal conditions, light touch sensory thresholds appear similar in both PAE and Sac offspring with hindpaw responses occurring at approximately 10 g of touch stimuli. In uninjured rats (sham treatment), sensory thresholds remained near their BL values on days 3 and 10 indicating that repeated stimulation to the hindpaws during von Frey behavioral testing does not affect threshold responses. In addition, following minor sciatic nerve 1-suture CCI in Sac rats, ipsilateral hindpaw responses remained close to their BL values on day 3, with sensory thresholds measured on day 10 that completely overlapped Sham-treated rats. However, striking allodynia was observed as measured by increased sensitivity of the ipsilateral hindpaw on days 3 and 10 after surgery (Fig. 1a). While threshold responses of the contralateral hindpaw remained stably near BL values in Sham-treated and Sac - 1 suture CCI rats, PAE rats with 1 suture CCI revealed a subtle but significant increase in contralateral hindpaw thresholds on days 3 and 10 (Fig. 1b). Thus, these initial data support that the enduring pathological consequences of PAE are revealed only after a minor nerve injury, where allodynia was clearly observed in 1-year aged rats. These data indicate that PAE creates long-lasting susceptibility to developing neuropathies, such as allodynia, following minor challenges to the nervous system.

### PAE results in long-term potentiation of allodynia following a standard chronic constriction injury

Using a standard 4-suture sciatic chronic constriction injury (CCI), we have previously reported that prenatal alcohol exposure (PAE) potentiates allodynia in young adult rats (4 months) compared to the levels of allodynia observed in saccharin (Sac) control offspring [25]. The current data demonstrate that the susceptibility to standard injury persists into middle age (12–13 month-old rats). BL thresholds in both PAE and Sac-treated rats were similar prior to surgery replicating data shown in Fig. 1a, b and prior work by Noor et al. [25]. Following standard CCI, Sac rats develop typical bilateral allodynia whereas PAE rats develop potentiated bilateral allodynia (Fig. 1c, d), also replicating our prior report in younger (4 month) adult rat offspring. Overall, these findings suggest that the effects of PAE are long-lasting and result in enhanced allodynia following a standard CCI in middle-aged offspring.

### PAE underlies enhanced spinal expression of astrocytic GFAP in neuropathic rats

**Fig. 2:**
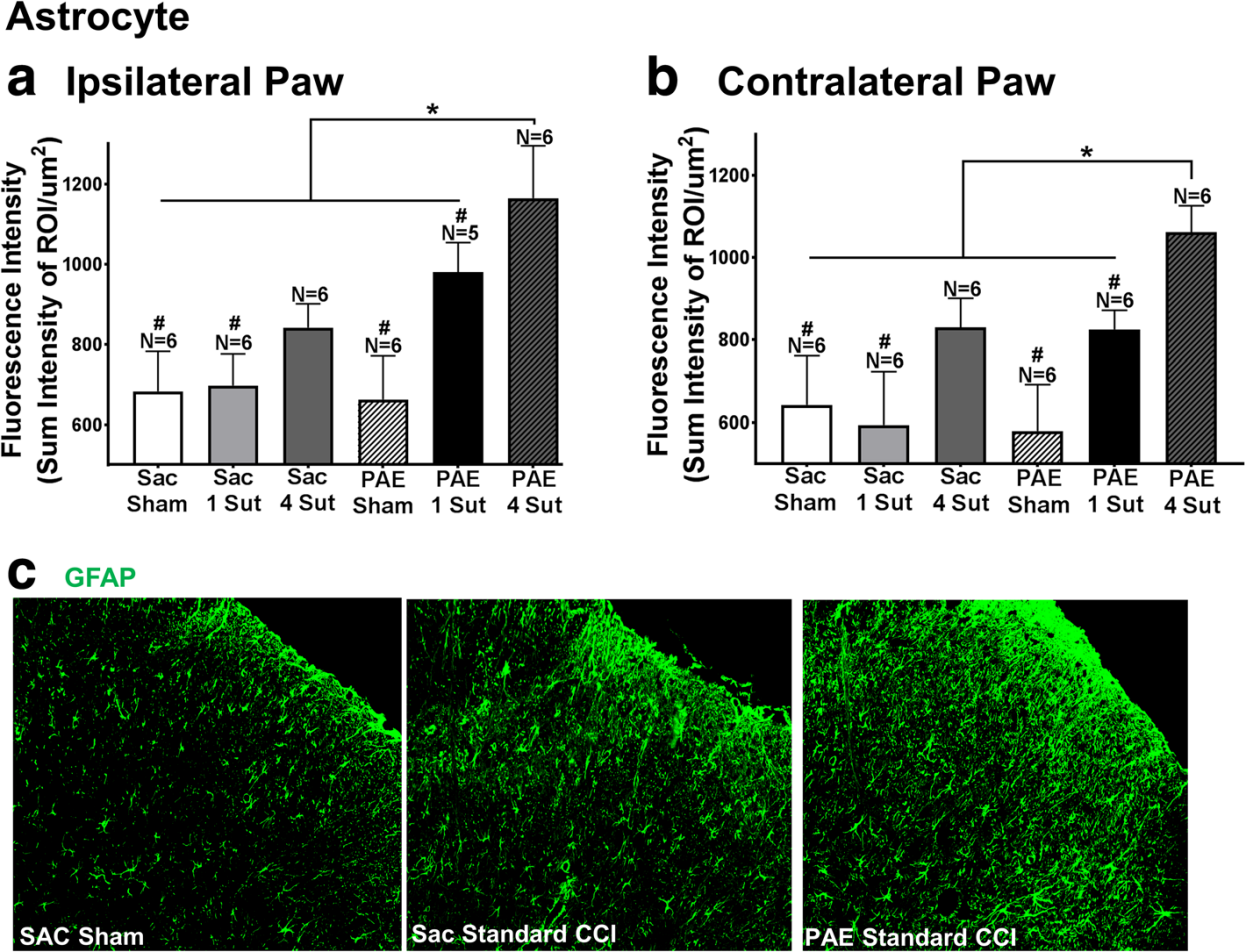
PAE induces heightened spinal cord astrocyte activation following both standard and minor CCI. Quantification of immunoreactivity (IR) following immunohistochemical (IHC) procedures was applied to assess the degree of astrocyte activation (GFAP) following standard and minor CCI. Fluorescence intensity is defined as the total sum intensity within the dorsal horn spinal cord (identified as a region of interest (ROI)) divided by the area of the ROI. **a**, **b** A main effect of alcohol exposure [ipsilateral: F_1,29_ = 36.62, p < 0.0001; contralateral: F_1,30_ = 17.35, p = 0.0002] and surgery [ipsilateral: F_2,29_ = 36.03, p < 0.0001; contralateral: F_2,30_ = 38.76, p < 0.0001] is seen in both ipsilateral and contralateral dorsal spinal cord relative to the side of sciatic nerve injury. PAE rats with standard 4-suture CCI had significantly elevated ipsilateral and contralateral GFAP IR compared to Sac control rats with standard 4-suture CCI. Following minor CCI, PAE rats had elevated ipsilateral and contralateral GFAP IR compared to PAE Sham, Sac Sham, Sac 1-suture. Minor CCI-PAE rats revealed comparable IR to Sac 4-suture. Representative images (**c**) of GFAP IR used in IHC analysis are shown for Sac Sham, Sac standard CCI, and PAE standard CCI conditions at 20x. *N* = 5–6 rats per group. Asterisks indicate *p* < 0.05. Number sign indicates significance amongst groups at *p* < 0.05. The data are presented as the mean ± SEM

Spinal astrocytes and microglia were evaluated in middle-aged Sac exposed or PAE offspring that had undergone standard or minor CCI. Behavioral assessment in these rats (data shown in Fig. 1) was terminated on day 10 post-surgery followed by spinal tissue collection and preparation for immunohistochemistry (IHC) to determine the immunoreactivity (IR) of the astrocyte “activation” marker glial fibrillary acidic protein (GFAP). Standard 4-suture CCI induced exaggerated astrocyte responses in both the ipsilateral and contralateral dorsal horn of the spinal cord relative to CCI in PAE rats that displayed bilateral allodynia compared to Sac rats (Fig. 2a, b). Minor/mild 1-suture CCI also resulted in significantly elevated bilateral spinal astrocyte responses in PAE rats compared to sham conditions and Sac rats with minor injury. It is notable that spinal cord dorsal horn GFAP IR from PAE rats with mild injury was comparable to Sac rats with standard CCI and allodynia (Fig. 1c and Fig. 2a). Representative images (Fig. 2c) of Sac-treated sham, Sac standard CCI, and PAE standard CCI conditions for GFAP used for analysis are shown. The results suggest that PAE underlies elevated astrocyte activation only following a secondary injury such as either standard or minor sciatic nerve injury.

### Elevated spinal microglial activation markers are only seen following standard CCI but not minor CCI

**Fig. 3:**
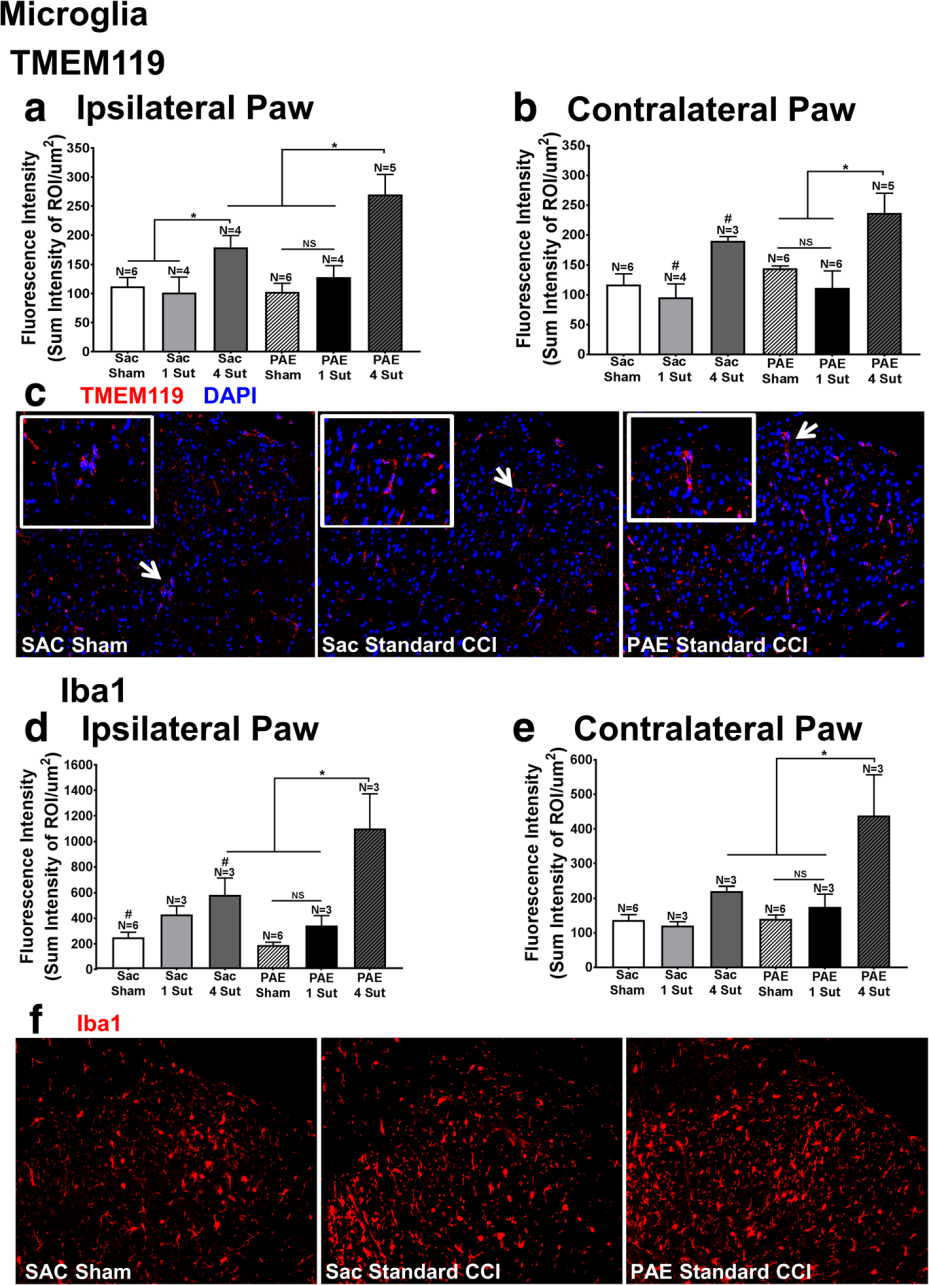
Minor CCI does not augment microglial activation in PAE rats. Applying IHC quantification following either standard CCI or minor CCI, two markers to identify alterations in microglial activation were examined: the microglial specific marker TMEM119 and the microglial and macrophage marker Iba1. **a**, **b** TMEM119 IR revealed a main effect of surgery [ipsilateral: F_2,23_ = 16.49, p < 0.0001; contralateral: F_2,24_ = 10.16, p = 0.0006]. Comparisons a priori of TMEM119 IR (**a**, **b**) showed that following standard CCI, PAE rats display elevated bilateral microglial activation compared to the PAE sham condition as well as PAE rats with minor injury. TMEM119 IR was also significantly elevated in PAE rats with standard CCI compared to Sac rats with standard CCI within the ipsilateral dorsal spinal cord. Sac rats with standard CCI displayed elevated TMEM119 IR within the ipsilateral dorsal spinal cord compared to Sac sham rats. These rats also revealed bilateral increases in TMEM119 IR compared to Sac rats with minor injury. No significant differences in TMEM119 IR were seen between PAE sham and PAE minor CCI conditions. **d**, **e** Iba1 results reveal a main effect of surgery [ipsilateral: F_2,18_ = 21.04, p < 0.0001; contralateral: F_2,18_ = 13.6, p = 0.0003]. Iba1 comparisons a priori (**c**, **d**) revealed significantly elevated IR in PAE with standard CCI compared to PAE sham rats, PAE rats with minor injury, as well as Sac rats with standard CCI. Sac rats with standard CCI had significantly elevated Iba1 IR compared to Sac sham rats in the ipsilateral dorsal spinal cord and contralateral spinal cord. No significant differences were seen between PAE sham and PAE minor CCI conditions. **e** Representative images of TMEM119 IR and **f** Iba1 IR that were included in the IHC analysis are shown for Sac Sham rats, Sac rats with standard CCI, and PAE rats with standard CCI at 20x. Inset for TMEM119 is shown at 60x. Asterisks indicate *p* < 0.05. NS indicates not significant. The data are presented as the mean ± SEM

Spinal dorsal horn microglial responses in middle-aged offspring following IHC processing revealed the transmembrane protein 119 (TMEM119), characterized as a microglial specific proliferation marker [31, 44], was significantly elevated bilaterally in PAE rats with standard CCI compared to PAE sham, PAE minor injury, and Sac standard CCI (Fig. 3a, b). Additionally, significant bilateral increases in spinal cord TMEM119 IR were observed in Sac rats with standard CCI compared to Sac offspring with sham treatment or minor CCI injury (Fig. 3a, b).

To provide parallel lines of evidence, we examined the expression of ionized calcium-binding adaptor molecule 1 (Iba1), a protein that is upregulated in both microglia and macrophages upon increased activation. Significant bilateral increases in Iba1 IR from Sac and PAE offspring were observed in the spinal cord dorsal horn with standard injury compared to all sham-treated rats, Sac minor and standard CCI and PAE with minor CCI. (Fig. 3d, e). Images of TMEM119 (Fig. 3c) and Iba1 (Fig. 3f) represent Sac sham, Sac standard CCI, and PAE standard CCI conditions. Insets for TMEM119 provide a representation of microglia surrounding nearby nuclei. Overall, these results suggest microglia may not be the key cell type responsible for primed spinal cord responses in PAE that lead to enhanced susceptibility to allodynia (Fig. 1c, d). It should be noted that our 7-μm tissue sections do not provide the thickness needed to display the multi-level microglial branching processes that TMEM119 IR has previously shown [44]. However, thin 7-μm sections allow for adjacent sections within the same anatomical regions of interest to be stained for different cellular markers. Additionally, decalcified spinal cords described above, underwent prolonged tissue decalcification that resulted in altered integrity of the Iba1 epitope. As a result, data for Iba1 IR from decalcified tissue was not included in the final Iba1 data set (Fig. 3c, d), as virtually no IR signal was detectable. To avoid artificially losing Iba-1 IR, a separate group of spinal cord tissue that did not undergo decalcification were isolated as described above. Subjects were removed if values fell 2 standard deviations ± mean. *N* = 2 subjects were removed. A total of 7 subjects (*N* = 2 Sac 1-Sut, *N* = 2 Sac 4-Sut, *N* = 2 PAE 1-Sut, *N* = 1 PAE 4-Sut) were removed from the ipsilateral data set and 6 subjects (*N* = 2 Sac 1-Sut, *N* = 3 Sac 4-Sut, *N* = 1 PAE 4-Sut) were removed from the contralateral data set in Fig. 3a, b.

### PAE alters the peripheral immune cell profile in middle-aged offspring

**Fig. 4:**
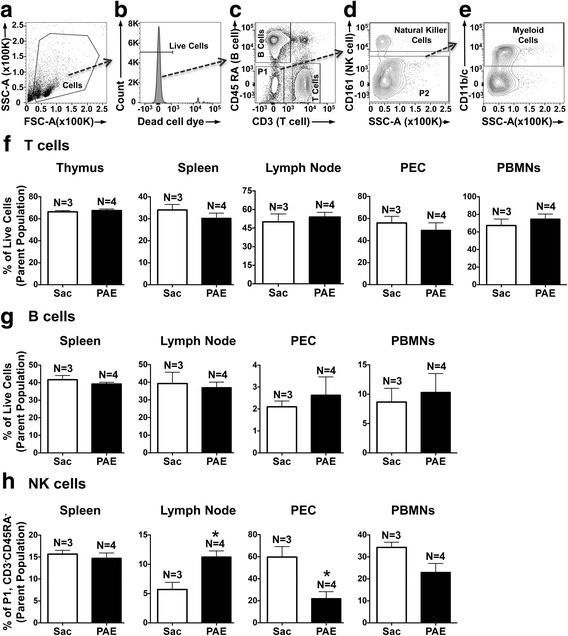
Characterization of major immune cell subsets in peripheral leukocytes as a consequence of PAE. **a**–**e** Representative flow cytometry plots and gating strategy to identify immune cell subsets in surgically naive Sac and PAE rats. **a** The intact/healthy cells were identified based on their light scatter properties. **b** Only live cells (verified by the absence of viability dye) were included for subsequent analyses. **c** T cells and B cells were identified by positive expression of CD3 and CD45RA, respectively. The double negative cell population was identified as P1. **d** Within P1, NK cells were quantified by their expression of CD161. The cell population not expressing CD161 was identified as P2. **e** P2 cells were further analyzed for the expression of CD11b/c (binds common epitope between CD11b and CD11c) to identify myeloid cells. Bar graphs represent proportions of (**f**) T cells, (**g**) B cells, and (**h**) NK cells in leukocytes collected from the thymus, spleen, lymph nodes, peritoneal cavity (PEC), and peripheral blood (PBMNs). Data are presented as percentages of their parent populations. A significant increase of NK cells was observed in the lymph node [t(5) = 4.028, *p = 0.01], whereas NK cells in the peritoneal cavity were reduced [t(5) = 3.349, *p = 0.02]. Data are representative of two independent experiments. Sac (*N* = 3 rats) and PAE (*N* = 4 rats), in each experiment (total of Sac; *N* = 6 and PAE; *N* = 8). Asterisks indicate p < 0.05. The data are presented as the mean ± SEM

In addition to spinal astrocytes and microglia, peripheral leukocytes, known to traffic to the level at the spinal cord where incoming nociceptive signals from damaged peripheral nerves communicate to pain projection neurons, may be important contributors of neuropathic pain. Our prior work revealed that PAE potentiates peripheral immune responses from the sciatic nerve following nerve injury in young adults. Thus, the phenotype of peripheral leukocytes in specific immune organs prior to trafficking to the spinal cord were examined for elevations in their specific populations or activation markers as an indication of a primed state at basal levels. Thus, general immune cell populations in the primary lymphoid organ (thymus) and secondary/peripheral lymphoid organs (spleen, lymph node) in middle-aged PAE offspring were characterized. In addition, immune cell composition was characterized in the circulating/peripheral blood and the peritoneal cavity. The major immune cell subsets, T cells, B cells, NK cells, and myeloid cells in these immune compartments were identified using the gating strategy represented in Fig. 4a–e. In the thymus, the majority of viable cells were mature T cells (Fig. 4f) with no differences between cells collected from Sac or PAE rat offspring. Note that the thymus is primarily involved in T cell maturation; therefore, it was not unexpected that other immune cell subsets were not reliably detected in the thymus (data not shown). T cells (about 30–65%) and B cells (2–40%) were identified in the spleen, lymph node, PECs, and the peripheral blood (Fig. 4f, g) with no differences in their proportions from Sac or PAE-treated rat offspring. Interestingly, compared to Sac-treated rats, NK cells collected from PAE rats were significantly increased in the lymph node, whereas the NK cell population was significantly decreased in the PECs (Fig. 4h). While there was a strong trend toward decreased NK cells in the PBMNs, the proportion of NK cells in the spleen from PAE and Sac rats were virtually the same (Fig 4h). Combined, these data suggest that of the immune cell examined, NK derived from PAE cells are somewhat primed under basal conditions and may contribute to enhanced trafficking following a secondary challenge, as they have already migrated to tissue areas where antigen presentation of pathogen- or danger-associated molecular patterns takes place.

### Myeloid cell proportions from middle-aged PAE offspring are elevated in secondary lymphoid organs

**Fig. 5:**
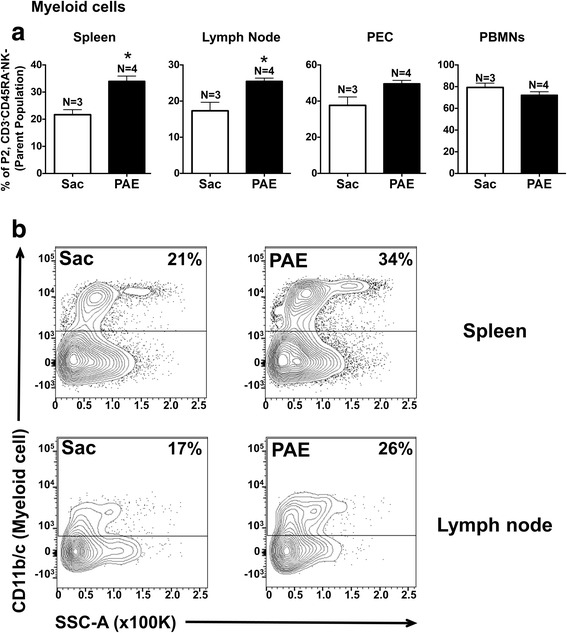
PAE induced myeloid cells in the secondary lymphoid organs. Leukocytes displaying negative expression of B cell, T cell, and NK cell markers (P2 population in Fig. 4) were further analyzed for myeloid cell marker (CD11b/c) expression. **a** Bar graphs showing proportions of myeloid cells in the spleen, medial iliac lymph nodes, peritoneal cavity (PEC), and peripheral blood mononuclear cells (PBMN). Myeloid cell proportions were significantly increased in the spleen [t(5) = 4.49, **p=0.0065]  and in the lymph nodes [t(5) = 3.704, *p=0.0139] in PAE rats. **b** Representative flow cytometry plots of CD11b/c expression vs side scatter (SSC-A) in the spleen (top panels) and in the lymph node (bottom panels) from Sac and PAE rats is provided. Data are representative of two independent experiments. Sac (*N* = 3 rats) and PAE (*N* = 4 rats), in each experiment (total of Sac; *N* = 6 and PAE; *N* = 8). Asterisks indicate *p* < 0.05. The data are presented as the mean ± SEM

The composition of myeloid immune cells was compared from the immune compartments, the spleen, lymph node, PECs, and PBMNs using the gating strategy described in Fig. 4a–e. Compared to Sac offspring, data revealed significant increases in myeloid cell proportions in the spleen and the lymph node of PAE rats (Fig. 5a, b for representative plots). More importantly, though CD11b/c expression is considered a general myeloid cell marker, expression and upregulation of CD11b/c often corresponds with leukocyte activation. Therefore, these data suggest a chronic basal increase in the programming of myeloid cell activation in peripheral lymphoid organs that occurs as a consequence of PAE. While the proportions of myeloid cells in the PBMNs and PECs were comparable between Sac and PAE groups (Fig. 5b), these data further support that immune cell responses in later life may be heightened following minor challenges.

### PAE primes peripheral immune cells resulting in augmented pro-inflammatory cytokine production

**Fig. 6:**
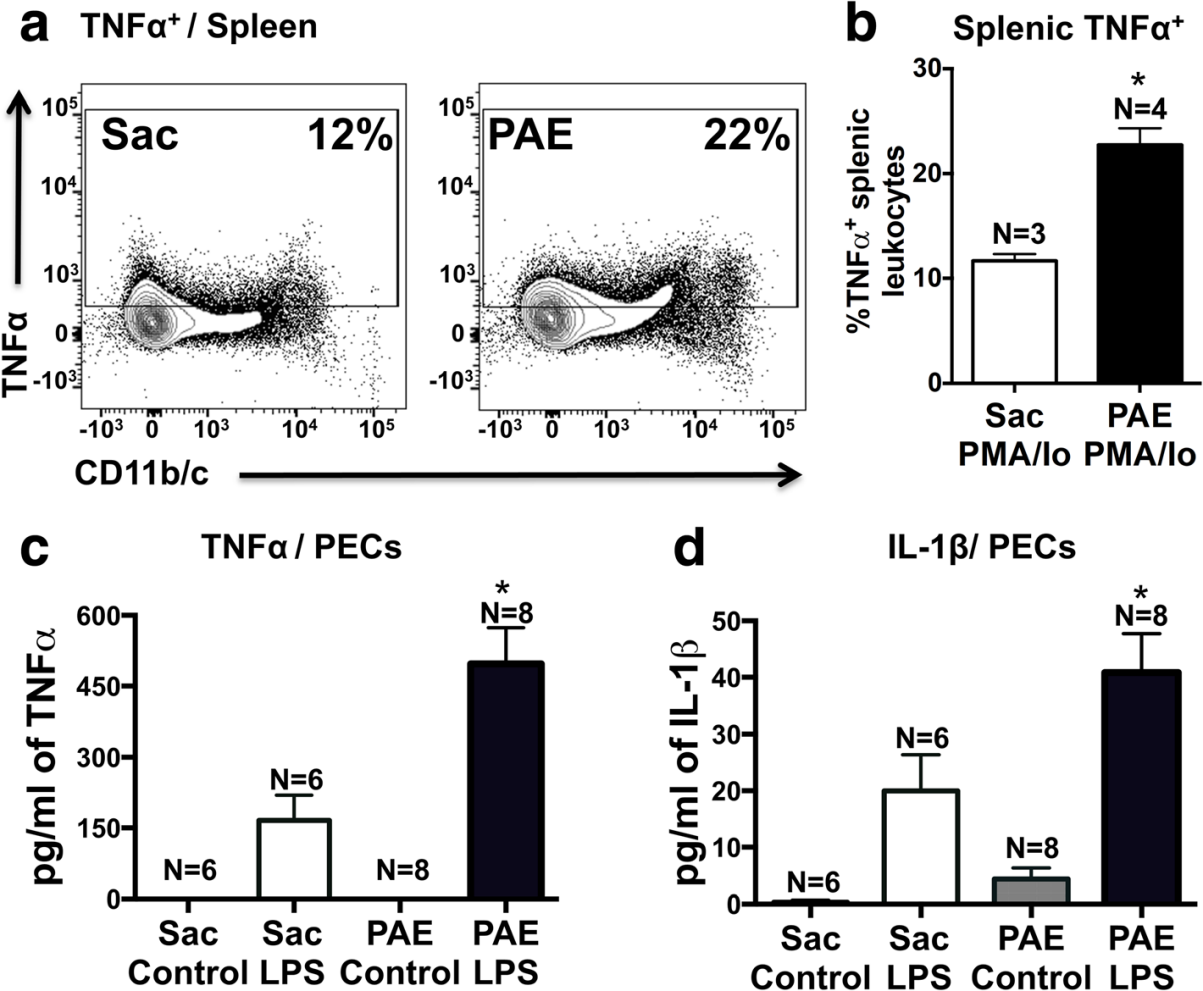
Peripheral immune cells in middle-aged PAE rats display augmented pro-inflammatory cytokine production. **a**, **b** Splenic leukocytes and **c**, **d** PEC were stimulated in vitro to examine their functional response profiles in proinflammatory cytokine production from Sac vs PAE rats. **a** Representative flow cytometry plots showing TNFα expression with CD11b/c expression on splenic leukocytes. **b** Bar graph of proportions of leukocytes displaying intracellular TNFα protein levels following 5 h of PMA/Io stimulation. Proportions of TNFα producing splenic leukocytes were significantly induced in PAE rats [t(5) = 5.6, *p = 0.0025]. , **c** TNFα levels in the media/cell-free supernatant after 24 h of LPS stimulation of PECs. Both LPS stimulation [F_1,24_ = 43.13, p <0.0001]  and PAE [F_1,24_ = 10.74, p = 0.0032]  increased TNFα production. A significant interaction between LPS stimulation and PAE was observed [F_1,24_ = 10.74], *p* = 0.0032]. LPS stimulated leukocytes from PAE rats induced the greatest amount of TNFα production than LPS stimulated leukocytes from Sac rats. **d** IL-1β levels in cell lysates of PECs following leukocyte stimulation with LPS. Both LPS stimulation [F_1,24_ = 31.85, p <0.0001] and PAE[F_1,24 _= 6.26, p = 0.0196] increased IL-1β production. Following stimulation with LPS, IL-1β production was significantly increased in leukocytes from PAE than leukocytes from Sac rats. *N* = 6 rats in Sac groups and *N* = 8 rats in PAE groups. Asterisks indicate *p* < 0.05. The data are presented as the mean ± SEM

The current report demonstrates alterations in peripheral immune cell populations (Figs. 4 and 5) of PAE rats that suggest the functional leukocyte responses to typical immune stimulators may be exacerbated to typical challenges. Leukocyte responses were evaluated in a mixed population of immune cells collected from Sac or PAE rats. PMA/Io (for splenocytes) and LPS (myeloid cells) stimulation was used to examine the protein production of the classic pro-inflammatory cytokines, TNFα and IL-1β, which are key in mediating allodynia following sciatic nerve damage. Data revealed a profound enhanced cellular response from 1-year-old PAE offspring compared to cellular responses from Sac offspring. Leukocytes were isolated from the spleen and peritoneal cavity. Following PMA/Io stimulation, significant increases in the proportions of TNFα producing splenic leukocytes were observed in cells from PAE compared to cells collected from the Sac group (Fig. 6a, b). Compared to leukocytes without LPS stimulation, LPS stimulation significantly induced TNFα and IL-1β protein levels in leukocytes from Sac offspring, as expected (Fig. 6c, d). However, LPS stimulation of leukocytes from PAE offspring resulted in the greatest increase in TNFα production as detected in the cell culture supernatant (Fig. 6c) and cell lysate (data not shown). Similarly, compared to stimulated leukocytes from Sac offspring, IL-β levels from cell lysates were greatest from PAE leukocytes stimulated with LPS (Fig. 6d). Together, these data demonstrate that adult immune cells that have previously undergone PAE appear “normal” under basal conditions, but generate exaggerated cytokine responses to typical stimuli. Moreover, the primed immune cell programming induced by PAE persists throughout adulthood. These data indicate the potential contribution of the PAE-primed peripheral immune system underlying the susceptibility to neuropathy in middle-aged rats.

## Discussion

Data from recent reports support the emerging idea that moderate drinking during pregnancy, which results in mean maternal peak serum ethanol levels of 60–80 mg/dL, exacerbates later-life central nervous system (CNS) dysfunction upon subsequent challenge either from immune activation or nervous tissue damage [25, 45]. These reports demonstrate that long-term PAE-related adverse outcomes ranging from cognitive deficits to potentiated peripheral neuropathy are associated not only with elevated CNS glial reactivity, but also adult-onset injury-induced exaggerated peripheral immune cell responses suspected to participate in ongoing peripheral neuropathies. A recent study using moderate PAE in rats conducted by Noor et al. [25] reported the striking observation of identical baseline sensory responses in saccharin and PAE offspring, suggesting the pathological consequences of PAE are not necessarily overt, but rather, can be unmasked following a secondary insult. These results further suggest PAE may create risks associated with later-life CNS pathology (i.e., neuropathy) triggered by challenges typically unnoticed and resolved in non-PAE individuals. The current work demonstrates that in later-life adult PAE offspring, PAE creates susceptibility to adult-onset peripheral neuropathy despite a substantial reduction in sciatic nerve injury not typically observed in the absence of prenatal alcohol exposure (Fig. 1).

In the current report, analysis of the dorsal horn of the spinal cord collected from the same rats examined for peripheral neuropathy revealed that standard (4-suture) CCI induces exaggerated bilateral microglial activation and proliferation, as assessed by immunoreactivity (IR) of two microglial markers Iba1 and TMEM119, providing converging lines of evidence (Fig. 3a–e). Elevated bilateral spinal astrocyte activation was observed concurrently, as measured by GFAP IR (Fig. 2). These data agree with previous reports that have demonstrated that both astrocytes and microglia mediate allodynia through their activation and release of proinflammatory cytokines [46]. Interestingly, spinal cords from PAE offspring with minor CCI (and corresponding allodynia) revealed no changes from sham conditions in microglial activation, as measured by both Iba1 and TMEM119, yet bilateral astrocyte activation was observed as significant increases in GFAP IR compared to Sac control rats (Fig. 2a, b) were evident. Together, these data suggest that spinal astrocytes and not microglia are playing a key role in mediating a PAE-associated increased risk for developing allodynia following minor injury. This notion is supported in previous studies showing no microglial activation in PAE animal within the visual cortex [47].

Further characterization of immune cell populations collected from surgically naïve saccharin and PAE offspring provided insight into the PAE-induced priming on immune subsystems, such as the adaptive immune system that requires the action of both B and T cells and the innate immune system that can initially act without T and B cell involvement [48]. Our T and B cell data revealed normal distribution patterns regardless of whether these cells were derived from Sac or PAE rats (Fig. 4f, g), which is consistent with prior reports [49–51]. However, other studies that ranged from a moderate PAE [22, 52] to a chronic life-long PAE [53] have shown cellular activation states and altered adaptive immune responses prior to subsequent challenge in adult rodents following PAE. The work in the current report support that further exploration into the possible effects of PAE on inflammatory versus anti-inflammatory T cell phenotypes underlying chronic neuropathy is needed. Using recently developed flow cytometry techniques, such as mass cytometry and imaging flow cytometry, would be beneficial [54–58].

Cellular populations critical to the function of the innate immune system revealed significantly different levels between Sac and PAE treatment groups (Fig. 4h). NK cells play a role similar to that assigned to T cytotoxic lymphocytes and provide rapid immune responses to tissue injury such as sciatic nerve damage, or infection [48]. NK cells populate/migrate to lymph nodes when activated and may influence adaptive immune cell differentiation [59, 60]. Thus, observing elevated NK cell populations in PAE lymph nodes suggests these cells are in a state of increased activation after trafficking from peripheral regions where they may reside in less differentiated and activated states.

Myeloid cell proportions were heightened in PAE rats specifically in the spleen and lymph node compared to Sac controls (Fig. 5), further supporting an increase in an activated phenotype. In general, myeloid cells act as potent antigen-presenting cells and upon immune activation, which can occur following recognition of altered self-antigens (i.e., damaged sciatic nerve axons); they acquire a “mature” differentiated state and relocate to secondary lymphoid organs to induce T cell-mediated immune responses [61]. In light of what is currently understood about myeloid cell function, the data reported here support the idea that cells from PAE rats are in a state of chronic mild heightened activation in key immune tissue systems. Given reported data here show elevated immune surveillance, the functional significance of these alterations was further explored and revealed that following in vitro stimulation, PAE-derived leukocytes show significant increases in TNFα and IL-1β protein (Fig. 6). Together, these data suggest that PAE alters the distribution of immune cells as well as their functional reactivity to immune stimulators that persists into middle age in PAE rats. A second insult results in heightened immune responses inducing allodynia.

### Mild CCI unmasks the effects of PAE-induced allodynia

Our previous report [25] demonstrated that following a standard CCI, young adult animals displayed potentiated allodynic responses which corresponded with increased immune reactivity compared to non-PAE Sac controls. Therefore, a relatively robust insult or challenge was required in this study to examine the predictable alterations in spinal neuronal processing (allodynia) to ascertain whether heightened reactivity could occur as consequence of PAE. However, homeostatic dysregulation still remained a question. The novel findings in the current report demonstrate that PAE results in a chronic *susceptibility* to CNS disease because the homeostatic mechanisms required for an appropriate CNS response to mild challenges, which *typically go unnoticed*, are now no longer intact in PAE offspring. Thus, the current report demonstrates that mild CCI creates allodynic responses *only* in PAE animals due to dysregulated homeostatic responses. Moreover, data from the current report replicate prior work by Noor and colleagues [25] showing that following PAE, sensory profiles and immune dysregulation are not observed under basal conditions (Figs. 1, 2, 3, 4, and 5). However, while standard CCI produces an upregulation of glial (both microglia and astrocytes) markers in both PAE and Sac rats, increased spinal astrocytes from PAE treated animals are observed following minor CCI rather than a standard CCI (Fig. 2). Additionally, ex vivo stimulation of leukocytes from PAE animals generated enhanced IL-1β and TNF-α cytokine responses (Fig. 6). Unexpectedly, minor CCI did not induce upregulations of spinal microglia (Fig. 3).

### Transmembrane protein 119 (TMEM119) is upregulated following standard, but not minor CCI

Transmembrane protein 119 (TMEM119) has previously been characterized to be involved in mouse osteoblast and stem cell induction and differentiation [62]. Furthermore, TMEM119 is expressed in the bone tissue such as the perichondrium and the trabecular bone [63]. Within the CNS, recent studies have shown that TMEM119 is expressed only in microglia [31]. Moreover, TMEM119 expression levels are increased following proliferation but not following stimulation using proinflammatory cytokines [31]. In support of a large body of evidence that spinal microglia are key mediators of pathological pain [10], TMEM119 transcripts within the spinal cord dorsal horn are upregulated in allodynic mice compared to controls [64]. Thus, this prior work supports the prediction that upregulation of TMEM119 corresponds to an increased proliferation of microglia involved in the induction of allodynia following a standard CCI (Figs. 1c, d and 3a, b). However, the absence of TMEM119 expression following minor CCI (Fig. 3a, b) observed in the current report further supports the hypothesis that microglia may not be a key mediator of PAE-induced allodynia from mild challenges.

### The effects of PAE are unmasked following a secondary insult

The results of these data may extend beyond neuropathic pain and may apply to other peripheral inflammatory and CNS conditions. Additionally, the long-term clinical implications of these results suggest that some adverse consequences of in utero alcohol exposure only occur upon a secondary insult sometime after prenatal alcohol exposure. This possibility is supported by studies in animal models of PAE where enhanced severity and disease outcomes are associated with influenza virus [53], adjuvant-induced arthritis [22], or following sciatic nerve damage in young adult PAE animals that display heightened allodynia and exaggerated glial activation [25]. Clinically, children with FASD display alterations in peripheral immune responses and show overall increased susceptibility to developing infections [23]. Collectively, these reports provide intriguing evidence that the effects of PAE on adult-onset disease are not observed until a secondary insult.

### PAE may increase susceptibility to neuropathic pain through alteration of the glutamate transporter, GLAST

Astrocytes have been well-characterized to act as key mediators of enduring allodynia following peripheral neuropathy [14, 65, 66], and this possibility is further supported by current data from PAE rats with allodynia. Prior work demonstrated that moderate PAE downregulates the expression of the astrocyte specific glutamate transporter, glutamate aspartate transporter or GLAST [67, 68], which may underlie the observed elevation in spinal astrocyte activation in PAE allodynia rats described in the current report. Indeed, following CCI in rats, spinal astrocyte-specific glutamate transporters downregulate leading to the induction of allodynia [69], which is thought to occur as a consequence of reduced glutamate clearance and enhanced glutamate action on spinal pain projection neurons. In addition, prior reports have shown that inhibition of astrocyte glutamate transporters results in potentiated allodynia [70]. Together, these data suggest that PAE-induced allodynia may be a result of alterations within astrocyte-specific glutamate transporters.

### Astrocyte and/or microglial activation is needed to induce bilateral allodynia from standard CCI

In PAE rats with minor injury, astrocyte but not microglial activation in the contralateral dorsal horn of the spinal cord are significantly elevated compared to sham conditions, coinciding with slight increases in contralateral hindpaw sensitivity. Previous publications suggest that contralateral allodynia is a result of astrocyte activation and subsequent release of proinflammatory cytokines [46] that spread to the contralateral spinal cord via communication through astrocyte-specific gap junctions [71]. Thus, it is possible that following minor CCI in PAE rats, elevated contralateral spinal cord astrocyte activation is sufficiently elevated to induce small increases in contralateral hindpaw sensitivity. This data supports the notion that a sufficient threshold of astrocyte activation is needed to induce bilateral allodynia. It should be noted that microglial activation is also observed following standard CCI. Previous reports have suggested that elevated microglial activation plays a greater role in the induction of contralateral allodynia [72]. Therefore, a combination of both astrocyte and microglial activation may be required to generate robust bilateral allodynia observed following standard unilateral sciatic nerve injury.

### PAE may lead to hyperalgesia following binge drinking in adulthood

It has been shown that third trimester prenatal alcohol exposure is able to induce a heightened pain response, clinically termed hyperalgesia, that is also seen during adulthood [73]. Hyperalgesia is a distinct pain-related phenomenon from allodynia because nociceptive-specific fibers are the necessary peripheral sensory fiber type responsible for “pain” relays. Hyperalgesia is the amplification of nociceptive signals (i.e., increased painful heat or painful pressures), whereas allodynia requires the action of non-nociceptive sensory fibers that results from miscoded non-painful stimuli as painful. Interestingly, other studies exploring PAE have shown that withdrawal during third trimester alcohol exposure is associated with increased glial activation and increased proinflammatory cytokine expression [16]. Note that glial cytokines are critical mediator allodynia. Previous studies examining animal models of drinking in adulthood have shown that withdrawal of ethanol intake is associated with induced hyperalgesia [74]. Additionally, chronic ethanol administration during adulthood was associated with increased expression of IL-1β and TNF-α following a secondary insult [75]. These studies in combination with the current report suggest that PAE primes the immune system, and following a secondary insult such as binge drinking may result in activation of glial-derived cytokine expression leading to hyperalgesia. Future studies will examine binge drinking in PAE and how it may lead to allodynia.

### Sex differences in immune regulation during FASD and neuropathic pain

Studies characterizing sexual dimorphic effects in PAE have found differences in the hypothalamic-pituitary-adrenal (HPA) axis [76], which is known to have regulatory effects on the immune system [77]. Furthermore, the acute inflammatory effects following LPS stimulation (< 24 h) of moderate PAE were found to be sex-specific [45]. Other studies in PAE have shown that males display a deficit in T cell proliferative responses compared to females [78]. Sex differences have also been shown in neuropathic pain studies, which demonstrated that the induction of neuropathic pain males and females are mediated by different neuroimmune mechanisms in [79]. Specifically, non-PAE males require both astrocytes and microglia to establish hypersensitivity whereas females utilize primarily T-cells [79, 80]. Ongoing studies aim to determine whether sex differences are present in PAE rats during the induction and maintenance of allodynia.

### FASD has similarities to other developmental disorders

Interestingly, many clinical similarities have been observed between FASD and autism spectrum disorders (ASD) as well as attention deficit hyperactivity disorder (ADHD) [81–83]. Moreover, from a behavioral standpoint, many characteristics seen in autism are also observed in FASD such as social impairment [82], cognitive deficits [84, 85]**,** and sensory impairments [7, 86]. Additionally, children with FASD have a high incidence of ADHD [87] where many social issues such as hyperactivity and impulsivity are observed [88]. Interestingly, as with FASD, many children with autism and ADHD also demonstrate an increased sensitivity to light touch [89, 90]. From an immunological standpoint, studies have found that autistic children display alterations in lymphocyte function [91] as well as an increased expression of cytokines such as IL-1β [92]. Other reports reveal that ADHD is closely related to an increased risk for allergic rhinitis [93] and upper respiratory infections [94], suggesting a potential immune dysfunction is concurrent with behavioral manifestations of these developmental deficits. Combined, these data suggest that the tactile dysfunction seen in ASD and ADHD may be due to similar underlying immune alterations observed in FASD.

## Conclusions

In conclusion, the risks of PAE associated with pathological pain endure long into middle adulthood and can be unmasked following even minor injuries to the peripheral nervous system. A contributing factor in the PAE-induced susceptibility to peripheral neuropathy is heightened spinal astrocyte responses following minor CCI. Additionally, not only does PAE alter peripheral immune cell distribution but also primes peripheral immune cell responses to immune stimuli resulting in heightened cytokine production. Heightened immune-cytokine responses as a consequence of PAE may be the critical underlying factor rendering one susceptible to chronic adult-onset neuropathies from mild insults. The results demonstrate that some of the adverse effects of PAE occur via long-lasting pathologically altered neuroimmune responses.

## Abbreviations

BL: Baseline
CCI: Chronic constriction injury
CNS: Central nervous system
DAPI: 4,6-Diamino-2-phenylindole
EDTA: Ethylenediaminetetraacetic acid
ELISA: Enzyme-linked immunosorbent assay
FASD: Fetal alcohol spectrum disorder
FBS: Fetal bovine serum
GFAP: Glial fibrillary acidic protein
Iba1: Ionized calcium-binding adapter molecule-1
IHC: Immunohistochemistry
IL-1β: Interleukin-1β
IR: Immunoreactivity
LPS: Lipopolysaccharide
NDS: Normal donkey serum
NK: Natural killer
PAE: Prenatal alcohol exposure
PBMNs: Peripheral blood
PBS: Phosphate buffered saline
PEC: Peritoneal exudate cells
PFA: Paraformaldehyde
PMA/Io: PMA/ionomycin
RBCs: Red blood cells
ROI: Region of interest
Sac: Saccharin
TMEM119: Transmembrane protein 119
TNF-α: Tumor necrosis factor-α
